# The Silent Giant: Asymptomatic Renal Cell Carcinoma With a Level III Inferior Vena Cava Thrombus

**DOI:** 10.7759/cureus.114535

**Published:** 2026-08-14

**Authors:** Sumith Deep, Ravi Krishnappa, Ashima Puri, Winthrop Pereira

**Affiliations:** 1 Surgical Oncology, JSS Hospital, Mysuru, IND; 2 General Surgery, JSS Hospital, Mysuru, IND

**Keywords:** clear cell rcc, inferior vena cava (ivc) tumor thrombus, ivc thrombectomy, oncovascular surgery, radical nephrectomy, renal cell carcinoma (rcc), sorafenib, tyrosine kinase inhibitors (tkis) therapy

## Abstract

Renal cell carcinoma (RCC) with inferior vena cava (IVC) tumor thrombus represents a complex clinical entity associated with increased surgical complexity and significant prognostic implications. We report the case of a 49-year-old asymptomatic male patient who was incidentally diagnosed with a massive right renal tumor with a Level III IVC tumor thrombus on imaging. The patient underwent successful right radical nephrectomy with IVC thrombectomy without the need for cardiopulmonary bypass. Histopathological examination confirmed clear cell RCC, Fuhrman grade II, with malignant thrombus and negative surgical margins. The final pathological stage was pT3bNxM0, corresponding to Stage III disease. This case highlights the importance of accurate preoperative imaging, multidisciplinary surgical planning, and meticulous vascular control in achieving successful thrombectomy of a Level III IVC tumor thrombus without cardiopulmonary bypass.

## Introduction

Renal cell carcinoma (RCC) accounts for approximately 2%-3% of all adult malignancies. A distinctive feature of RCC is its propensity for venous extension, with tumor thrombus (TT) potentially extending into the renal vein or inferior vena cava (IVC), seen in approximately 4%-10% of cases [1]. Although the classical triad of flank pain, hematuria, and palpable mass is well described, it is seen in only a minority of patients, with many remaining asymptomatic until advanced stages.

The presence of venous TT is an important prognostic factor and is associated with increased surgical complexity and adverse outcomes. Accurate identification using contrast-enhanced computed tomography (CECT) or magnetic resonance imaging (MRI) is essential, as TT may be misinterpreted as bland thrombus if not carefully evaluated [2]. Despite aggressive surgical management, patients with high-risk features remain susceptible to recurrence, underscoring the need for continued evaluation of adjuvant therapeutic strategies in locally advanced disease.

## Case presentation

A 49-year-old male patient was referred to the surgical outpatient department for evaluation of infertility. During the evaluation, abdominal imaging incidentally revealed a large right renal mass, and the patient was subsequently referred to Surgical Oncology. The patient was asymptomatic with respect to the renal tumor at presentation.

On clinical examination, a right lumbar mass measuring approximately 16 × 12 cm was palpable. During hospitalization, the patient subsequently developed a persistent low-grade fever with no identifiable source of infection. Laboratory investigations, including total leukocyte count and urine analysis, were within normal limits.

**Figure 1:**
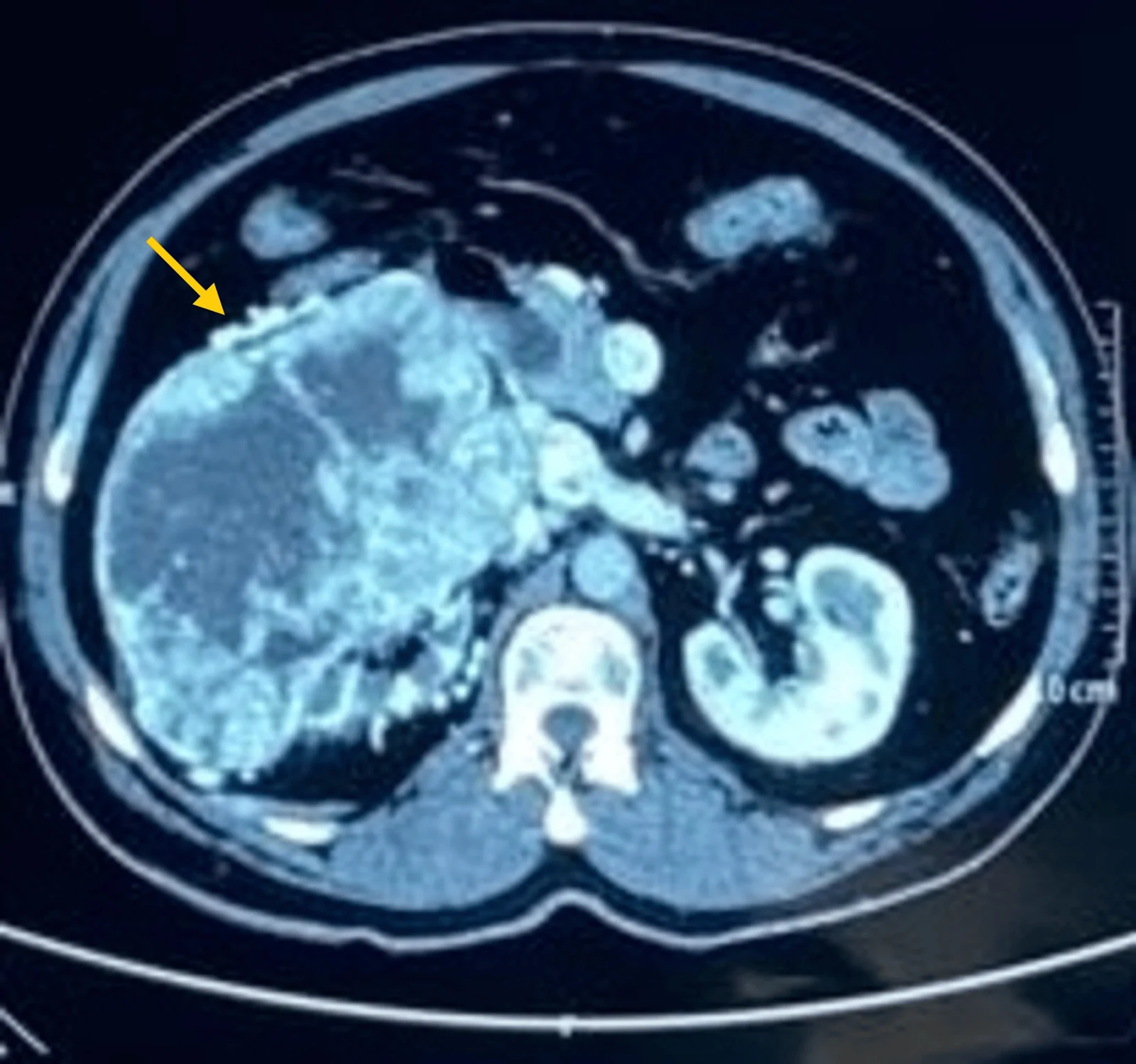
Preoperative axial contrast-enhanced computed tomography (CECT) of the abdomen and pelvis. The yellow arrow demonstrates a large right renal mass.

Initial ultrasonography revealed a large intra-abdominal mass in the right lumbar region. For further staging and anatomical delineation, CECT of the thorax, abdomen, and pelvis was performed. CECT demonstrated a heterogeneous right renal mass measuring approximately 18 × 16 cm arising from the right kidney, suspicious for RCC. There was no evidence of regional lymphadenopathy or distant metastases (Figure 1). Vascular evaluation revealed a Level III IVC TT according to the Neves and Zincke classification, characterized by extension into the retrohepatic segment of the IVC up to the level of the hepatic veins, without supradiaphragmatic or right atrial involvement [3].

With a diagnosis of locally advanced RCC with Level III IVC TT and no evidence of distant metastases, the case was discussed at a multidisciplinary tumor board, and upfront surgery was planned. The patient underwent right radical nephrectomy with IVC thrombectomy and patch closure of the IVC venotomy. The procedure was performed by a multidisciplinary team comprising Surgical Oncology and Cardiothoracic and Vascular Surgery.

**Figure 2:**
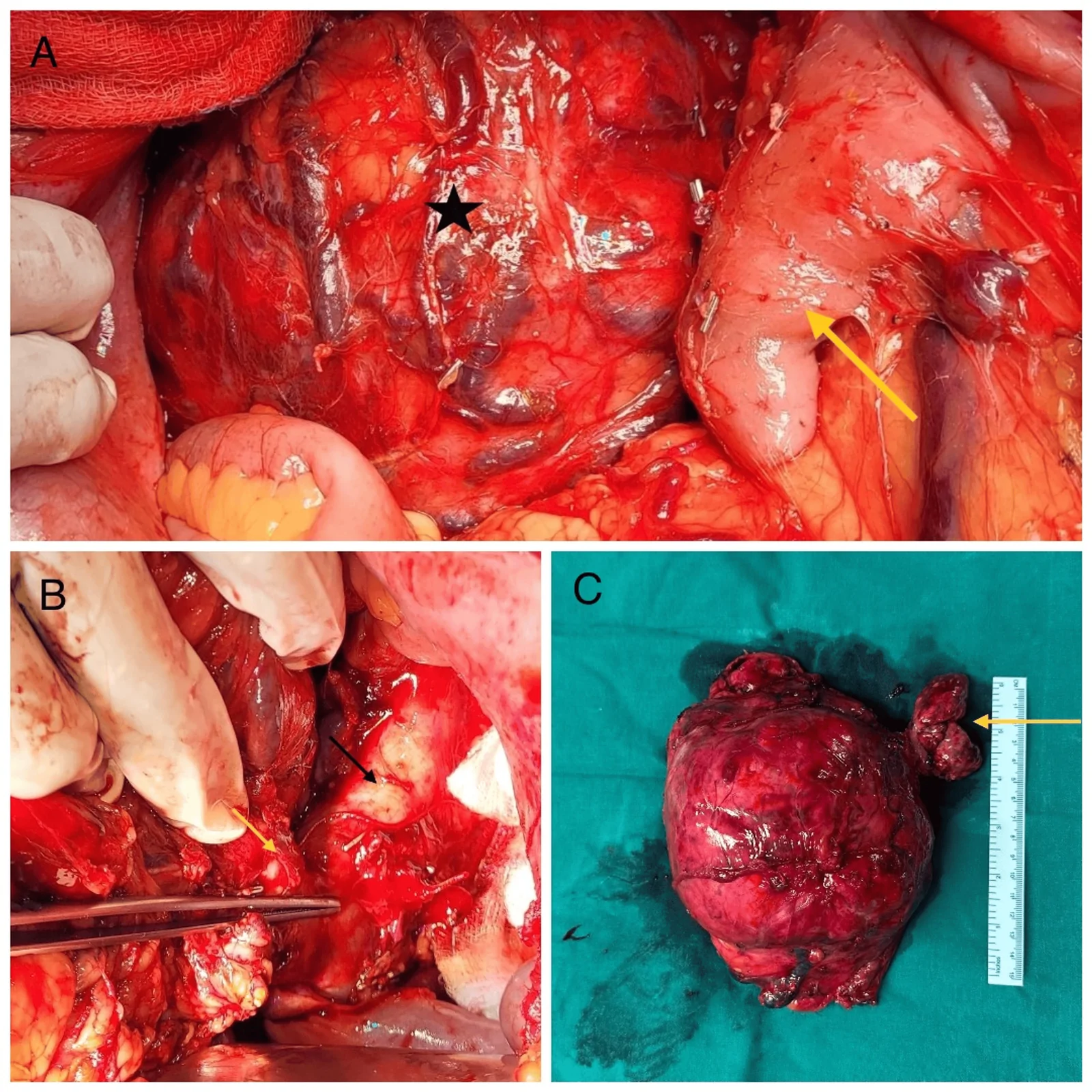
Intraoperative findings during right radical nephrectomy and inferior vena cava (IVC) thrombectomy. (A) Surgical exposure showing the right renal tumor and adjacent structures. Black star: right renal tumor; yellow arrow: Kocherized duodenum. (B) Intraoperative identification of vascular anatomy during IVC thrombectomy. Instrument: right renal vein-IVC junction; yellow arrow: right renal tumor; black arrow: infrahepatic IVC. (C) Gross postoperative specimen following radical nephrectomy and IVC thrombectomy. Yellow arrow: right renal cell carcinoma (RCC) with IVC tumor thrombus, resected en bloc.

Intraoperatively, a well-circumscribed mass measuring approximately 18 × 16 cm was identified arising from the right kidney, extending superiorly into the subhepatic space, abutting the IVC medially and adherent to the abdominal wall laterally (Figure 2A). Following careful mobilization of the right renal tumor, the renal artery was controlled early to facilitate further tumor mobilization and reduce venous congestion. Hepatic mobilization was then performed to facilitate exposure of the retrohepatic IVC and hepatic veins (Figure 2B). The IVC was dissected proximally and distally to the TT, with identification and preservation of the contralateral renal vein. The relationship of the thrombus to the hepatic veins was assessed, and proximal and distal vascular control was secured.

The IVC was subsequently clamped, and a controlled cavotomy was performed. The TT was carefully extracted through the cavotomy without fragmentation. The IVC lumen was inspected to ensure complete thrombus clearance, following which the venotomy was closed using a Teflon patch. The vascular clamps were released after ensuring adequate hemostasis and satisfactory caval flow. The radical nephrectomy was then completed, and the specimen was removed (Figure 2C).

The total IVC clamp time was 15 minutes, with an estimated blood loss of 800 mL. The procedure was completed without the need for cardiopulmonary bypass. Postoperatively, the patient was monitored in the intensive care unit for two days and subsequently transferred to the ward. The total postoperative hospital stay was six days. The postoperative course was uneventful, with no significant hemorrhage, lower limb edema, or thromboembolic complications. Renal function remained stable, and the patient was discharged in a clinically stable condition.

**Figure 3:**
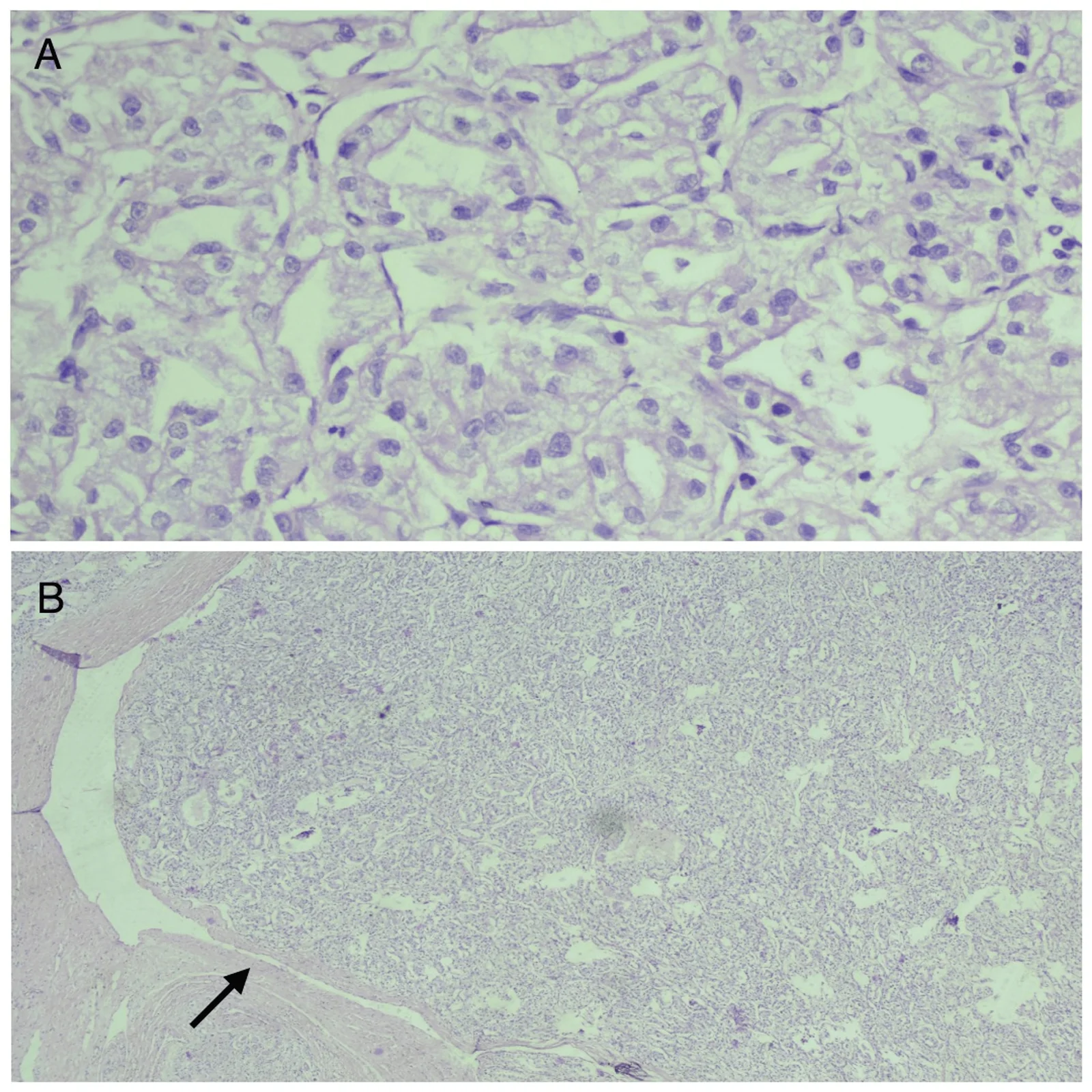
Histopathological examination of clear cell renal cell carcinoma. (A) H&E stain, x40 magnification: the section shows tumor cells arranged in sheets, separated by fibrovascular septae. Individual tumor cells have vesicular nuclei, prominent nucleoli, and clear cytoplasm. (B) H&E stain, x4 magnification: section of renal vein showing malignant tumor thrombus attached to the venous wall (black arrow).

Histopathological examination confirmed clear cell RCC, reported as Fuhrman grade II. Surgical margins were free of tumor, and no lymphovascular invasion was identified (Figure 3A). The TT contained malignant cells, and sections from the renal vein demonstrated tumor tissue attached to the venous wall (Figure 3B). The disease was pathologically staged as pT3bNxM0 according to the American Joint Committee on Cancer (AJCC) 8th edition [4], corresponding to Stage III disease. The pathology report available for the case used Fuhrman grading; this terminology has therefore been retained rather than retrospectively assigning a WHO/International Society of Urological Pathology (ISUP) grade without the original pathological criteria.

Given the high-risk pathological features and the presence of venous TT, adjuvant systemic therapy was considered following multidisciplinary tumor board discussion. Pembrolizumab was considered but was not financially feasible for the patient. Following multidisciplinary discussion and consideration of the treatment options accessible to the patient in the prevailing clinical setting, sorafenib was selected and administered for one year.

**Figure 4:**
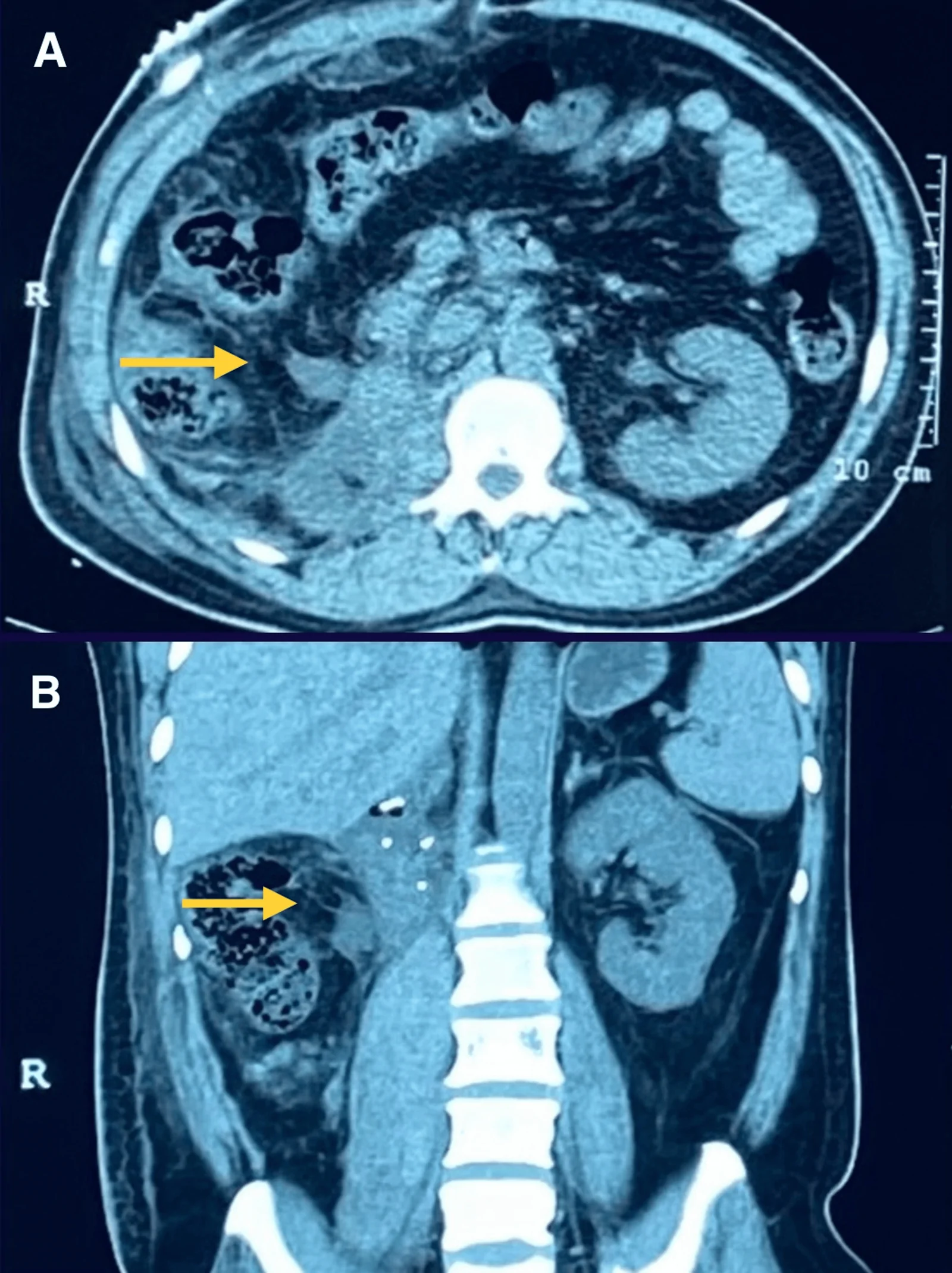
One-year postoperative contrast-enhanced computed tomography (CECT) reconstruction of the abdomen and pelvis. The yellow arrow demonstrates absence of the right renal tumor with no evidence of recurrence. (A) Axial section; (B) coronal section.

The patient completed one year of sorafenib and remained under close oncological surveillance, with alternating MRI and CECT of the abdomen at three-month intervals. At the one-year follow-up, the patient remained asymptomatic, with no evidence of recurrence on imaging (Figure 4). Renal function was preserved, and the patient maintained a good Karnofsky performance status.

## Discussion

Radical nephrectomy with IVC thrombectomy remains the cornerstone of management for resectable RCC with venous tumor extension. TTs are commonly classified according to the Neves and Zincke classification based on their cranial extent within the IVC: Level I: the TT extends less than 2 cm above the renal vein, reaching just into the IVC. Level II: the TT extends more than 2 cm above the renal vein but remains below the hepatic veins, occupying the infrahepatic IVC. Level III: the TT extends up to the hepatic veins but remains below the diaphragm, occupying the retrohepatic or intrahepatic IVC. Level IV: the TT extends above the diaphragm into the supradiaphragmatic IVC and may reach the right atrium [3].

The thrombus level directly influences operative planning and the requirement for advanced vascular control or cardiopulmonary bypass. In the present case, the thrombus was classified as Level III, extending to the level of the hepatic veins while remaining below the diaphragm, with no evidence of suprahepatic, supradiaphragmatic, or right atrial extension on preoperative imaging. This anatomical configuration permitted an infradiaphragmatic approach with controlled vascular exposure and thrombectomy without cardiopulmonary bypass.

For Level III thrombi, adequate exposure of the retrohepatic IVC and appropriate proximal and distal vascular control are essential. Liver mobilization and exposure of the retrohepatic IVC may be required depending on the precise relationship of the thrombus to the hepatic veins. Controlled cavotomy and removal of the TT without fragmentation are fundamental surgical principles. In selected patients, these techniques allow complete thrombectomy without cardiopulmonary bypass [5]. Avoidance of cardiopulmonary bypass may reduce the risks associated with extracorporeal circulation, including coagulopathy, systemic inflammatory response, and neurological complications [5].

A notable aspect of this case is the marked discordance between tumor burden and clinical presentation. Despite an 18 × 16 cm renal mass and significant venous extension, the patient was asymptomatic with respect to the renal tumor at presentation. The mass was detected incidentally during imaging performed as part of an infertility evaluation. This highlights the often clinically silent nature of RCC and the importance of incidental imaging in detecting advanced disease [1,6].

The patient developed a persistent low-grade fever only after hospital admission and had no identifiable infectious source. Although this may represent a paraneoplastic manifestation, this interpretation remains speculative. Paraneoplastic manifestations of RCC have been associated with tumor-derived cytokines, including interleukin-6 [7].

Preoperative imaging played a critical role in accurately delineating the renal mass, the extent of venous TT, and its relationship to the hepatic veins. Accurate distinction between TT and bland thrombus is particularly important because the two entities have different therapeutic implications [2]. In this case, the preoperative imaging findings correlated with the intraoperative extent of the thrombus and facilitated appropriate surgical planning.

The presence of IVC TT is associated with more advanced disease and poorer prognosis than localized RCC. Nevertheless, complete surgical resection remains an important determinant of outcome in appropriately selected patients, with reported five-year survival rates of approximately 40%-65% in selected surgically treated cohorts [5,8].

The role of regional lymphadenectomy in RCC remains controversial. Gershman et al., using a propensity score-based analysis, found no significant survival benefit associated with lymph node dissection in nonmetastatic RCC and suggested that its role may be primarily diagnostic rather than therapeutic. Therefore, lymphadenectomy is generally reserved for patients with clinically suspicious nodal disease or selected staging indications [9].

Beyond the technical aspects of surgical management, the high-risk nature of RCC with venous TT also warrants consideration of adjuvant systemic therapy, which has evolved considerably in recent years. Earlier studies evaluating tyrosine kinase inhibitors (TKIs) produced inconsistent results. The ASSURE trial did not demonstrate a survival benefit with adjuvant sunitinib or sorafenib, while the S-TRAC trial demonstrated improved disease-free survival with sunitinib without a corresponding overall survival advantage [10,11]. Similarly, the ATLAS and SORCE trials did not establish a consistent benefit for adjuvant targeted therapy [12,13]. These findings have limited the role of adjuvant TKIs in contemporary practice.

More recently, the treatment landscape has shifted toward immune checkpoint inhibition. The KEYNOTE-564 trial demonstrated a significant improvement in disease-free survival with adjuvant pembrolizumab in patients with clear cell RCC at increased risk of recurrence following nephrectomy [14]. Subsequent follow-up demonstrated a significant overall survival benefit, further supporting the role of adjuvant pembrolizumab in appropriately selected high-risk patients [15].

The present case therefore requires contextual interpretation of the adjuvant treatment decision. Although the patient’s pT3bNxM0 disease with venous TT placed him in a high-risk category for recurrence, pembrolizumab was considered but was not financially feasible. After multidisciplinary discussion and consideration of the treatment options available to the patient in the prevailing clinical setting, sorafenib was selected and administered for one year.

Importantly, sorafenib should not be presented as current standard-of-care adjuvant treatment for high-risk clear cell RCC. Its use in this case represents a context-specific therapeutic decision rather than evidence supporting the efficacy of adjuvant TKI therapy. The negative or inconsistent results of the major adjuvant TKI trials, together with the subsequent evidence supporting adjuvant pembrolizumab, demonstrate the evolution of systemic treatment for high-risk RCC [10-15].

At the one-year follow-up, the patient remained asymptomatic and had no radiological evidence of recurrence. This favorable short-term outcome represents the observed clinical course of this individual patient and should not be interpreted as evidence of sorafenib efficacy.

The management of RCC with Level III IVC TT requires careful integration of radiological assessment, surgical expertise, vascular control, and perioperative planning. In this case, the absence of distant metastases, the infradiaphragmatic extent of the thrombus, and the feasibility of obtaining adequate vascular control permitted radical nephrectomy and thrombectomy without cardiopulmonary bypass.

The multidisciplinary involvement of Surgical Oncology and Cardiothoracic and Vascular Surgery provided appropriate expertise for the management of this technically demanding case. The case also illustrates that management of high-risk RCC extends beyond technically successful surgery. Contemporary multidisciplinary care should incorporate evidence-based systemic therapy, with treatment decisions individualized according to oncological risk, eligibility, availability, affordability, and patient preference.

## Conclusions

This case highlights the potential for RCC with IVC TT to remain clinically silent despite an extensive tumor burden. Accurate preoperative imaging is critical for defining tumor and thrombus extent and for planning the surgical approach. Radical nephrectomy with IVC thrombectomy remains the cornerstone of treatment for resectable RCC with venous tumor extension, and selected Level III thrombi can be managed successfully without cardiopulmonary bypass using meticulous vascular control and appropriate multidisciplinary expertise.

The case also emphasizes the importance of integrating contemporary evidence-based systemic therapy into the multidisciplinary management of high-risk clear cell RCC. Although sorafenib was administered in this patient because pembrolizumab was not financially feasible, it should not be regarded as current standard-of-care adjuvant therapy. Current evidence supports consideration of adjuvant pembrolizumab in appropriately selected high-risk clear cell RCC following nephrectomy. Optimal management therefore requires both technically sound surgery and individualized, evidence-based systemic treatment planning.
